# System Dynamics Model and Simulation of Employee Work-Family Conflict in the Construction Industry

**DOI:** 10.3390/ijerph13111059

**Published:** 2016-10-28

**Authors:** Guangdong Wu, Kaifeng Duan, Jian Zuo, Jianlin Yang, Shiping Wen

**Affiliations:** 1 School of Tourism and Urban Management, Jiangxi University of Finance & Economics, Nanchang 330013, China; gd198410@163.com (G.W.); kefee920729@163.com (K.D.); yangjl_jxufe@sina.com (J.Y.); 2 School of Architecture and Built Environment, Entrepreneurship, Commercialisation and Innovation Centre (ECIC), The University of Adelaide, Adelaide 5005, Australia; jian.zuo@adelaide.edu.au; 3 School of Economics and Management, Tongji University, Shanghai 200092, China

**Keywords:** construction employees, work-family conflict, system dynamics, simulation

## Abstract

The construction industry is a demanding work environment where employees’ work-family conflict is particularly prominent. This conflict has a significant impact on job and family satisfaction and performance of employees. In order to analyze the dynamic evolution of construction industry employee’s work-family conflict between work and family domains, this paper constructs a bi-directional dynamic model framework of work-family conflict by referring to the relevant literature. Consequently, a system dynamics model of employee’s work-family conflict in the construction industry is established, and a simulation is conducted. The simulation results indicate that construction industry employees experience work interference with family conflict (WIFC) levels which are significantly greater than the family interference with work conflict (FIWC) levels. This study also revealed that improving work flexibility and organizational support can have a positive impact on the satisfaction and performance of construction industry employees from a work and family perspective. Furthermore, improving family support can only significantly improve employee job satisfaction.

## 1. Introduction

Work-family conflict has a negative impact on an employee’s work and family life, as well as his/her physical and mental health and well-being [1,2,3,4]. Similarly, work-family conflict leads to higher job stress levels and dissatisfaction levels, which can even lead to resignation [5,6,7]. In addition, work-family conflict has a significant negative impact on organizational strategy, performance and commitment, as well as the employee’s psychological commitment to the organization [8,9,10,11,12]. Work-family conflict has become a common concern of workplace stress research.

In the context of construction industry, the work-family relations of employees have been largely ignored [13]. Previous studies have shown that the demanding work environment of the construction industry has the potential to significantly interfere with the family and social role of employees [13,14,15], which makes the employee work-family conflict particularly prominent compared to other industries [16]. As a labor-intensive industry, the construction industry features high level of risks, heavy workload, and long project duration. These lead to long working hours, work overload and high work-family conflict for employees. The employees, however, play a vital role in achieving project objectives and project success. Hence, it is imperative to study these employees’ work-family conflicts and the relationship between work and family life.

In recent decades, work-family conflict has drawn growing public interest [17]. Despite diverse research methods used in previous studies, the mainstream research into work-family conflict is quantitative and empirical where questionnaire survey is involved [18]. However, the empirical research of social science has always been subjected to “data quality constraint” and “low level repetition” traps [19]. “Data quality constraint” means that the data quality requirements of the empirical social science research are very strict, any omissions or errors of the data source and the data processing may lead to fatal error in result or judgment. “Low level repetition” means that since there are many research models in the empirical research of social science for imitation and reference, the researchers tend to excessively pursue the form of repetition whereas the quality of the study is overlooked. The research methods employed in the field of work-family conflict should not be limited solely to quantitative verification. Rather, attention should also be paid to the diversity of research methods [18]. The variety of research methods can offset for the deficiencies associated with using the single research method [20]. This paper attempts to analyze the dynamic evolution of construction industry employee’s work-family conflict between work and family domains via a novel approach, i.e., system dynamics model simulation. By researching the existing literature, a bi-directional dynamic model framework of work-family conflict is developed. Consequently, the system dynamics model is established from the perspective of employee’s work-family relationships in the whole life cycle of construction projects. In addition, a model simulation is conducted, in order to identify the internal rules of the evolution of employee work-family conflict in work and family settings. Findings provide both theoretical reference and practical advice relating to work-family conflict in the construction industry.

## 2. Literature Review

### 2.1. Work-Family Conflict

Early research into work-family conflict (WFC) was mainly based on the role theory perspective. This theory regards work-family conflict as the stress resulting from the role conflict caused by individual’s incompatible work and family roles. Kahn et al. stated that work-family conflict is the conflict and pressure placed on an individual to convert between the two incompatible roles of work and family [21]. Greenhaus and Beutell first put forward the explicit definition of work-family conflict in 1985. They stated that work-family conflict is “a form of inter-role conflict in which the role pressures from the work and family domains are mutually incompatible in some respect”. They identified three forms of WFC, namely (1) time-based WFC; (2) strain-based WFC and (3) behavior-based WFC [22]. This concept has been adopted in the academic community as a means to form a basic consensus. Traditionally, WFC research was uni-directional; the focal point was strictly on the conflict which occurred when work interfered with family. However, when probing more deeply into this field of study, Gutek et al. found that each of the three forms of conflict proposed by Greenhaus and Beutell has two directions, namely work interfering with family and family interfering with work [23]. On this basis, Frone et al. proposed a bi-directional concept of WFC, thus dividing WFC into two types of conflict: (1) work interference with family conflict (WIFC) and (2) family interference with work conflict (FIWC) [24]. Previous studies have indicated that employees experience WIFC to a greater degree than FIWC [23,25].

### 2.2. The Antecedents of WFC

Throughout the existing research in the field of WFC, the antecedents of WFC can be summarized into the three aspects of (1) work domain; (2) family domain and (3) individual characteristics. Of these three aspects, variables from the work domain have a strong predictive effect on WIFC, while variables from the family domain have a strong predictive effect on FIWC. Individual characteristics include personality, emotion, values and other personal traits. As such, the WFC of different personality traits will also be different. In Netemeyer’s definition of WFC, job requirements, working hours and job pressures are important factors which hinder family life [25]. The greater the workload, the greater the job pressure, and the greater the likelihood that WIFC will increase. Byron indicated that variables from the family domain (e.g., family involvement and family role conflict) have higher degree of correlation to FIWC than the variables from work domain [26]. The social support theory suggests that social support from the employer and family domains is also an important factor affecting WFC. Carlson and Perrewé pointed out that family support (e.g., support from spouses, relatives and parents) helps to ease WFC [6]. Many following studies have confirmed this view [27,28,29]. At the same time, Anderson et al. proposed that organizational support can alleviate the negative consequences caused by WFC [30]. In addition, the boundary theory argued that work and family are two relatively independent domains which have their respective boundaries. However, the theory also stated that the boundary of the scope of each role has permeability and flexibility [31]. Many studies have found that work flexibility can improve the work-family balance [32,33], thus reducing the negative impact of WFC on work and family life. In this paper, job involvement, job pressure, family involvement and family pressure are selected as the antecedent variables of WFC, while organizational support, family support and work flexibility are chosen as adjustment variables.

### 2.3. The Outcome Variables of WFC

Organizational behavior researchers are concerned with the outcome variables of WFC. This concern stems from the fact that WFC has a significant impact on an employee’s work, family, and physical and mental health and well-being. WFC can specifically produce the three aspects of work-related, family-related and stress-related results. Existing literature indicates that WIFC and FIWC may both have a negative effect on the outcome variables of work and family. Eby pointed out that an increase in WFC can reduce employee job satisfaction and organizational commitment. In such circumstances, employee turnover intentions rise [34]. Karatepe and Sokmen’s study reported that WFC may be related to low family satisfaction and low marital satisfaction [35]. Frone et al. proposed that WFC not only reduces the quality of family life, but also leads to low job performance and low family performance [36]. Although some scholars have suggested that WIFC produces more negative results in terms of family, while FIWC produces more negative results in terms of work, other studies have shown that WIFC and FIWC have a cross domain relationship with the outcome variables in the work and family field [35]. In addition, WFC is related not only to employee depression, anxiety and other mental health indicators, but also to smoking, alcohol abuse and unhealthy eating habits. WFC can cause burnout and depression [37] and increase the risk of emotional exhaustion [38]. This paper chooses to study satisfaction and performance from the work and family domains as the outcome variables of WFC.

### 2.4. Research into WFC in the Field of Construction Engineering

The construction industry is considered to be a high-conflict [39] and high-pressure [40,41,42] work environment. Long working hours and work overload place great pressure on construction project staff [41,42,43]. Ng et al. found that WFC was one of the most difficult stressors for construction industry project professionals to manage [44]. Turner and Mariani pointed out that for construction industry workers, critical factors to time-based and strain-based WFC were: number of working hours, work-schedule fit, management support and flexibility [45]. Researchers in the Australian construction industry have explored the antecedents of WFC. For example, Lingard and Francis found that construction industry workers experience high levels of WFC, which can be predicted by excessive work demands (e.g., irregular and long work hours) [13]. Other studies into the construction industry showed that competitive tendering [46] and tight project planning [47] led to long working hours, which in turn impacted significantly on employee work-life stress. Further research indicated that working hours, supervisor support and job flexibility could affect the degree of employee conflict [48]. WFC acts as the connection mechanism between work-schedule demands and employee burnout [49], while organizational support and other support characteristics at work can ease the relationship between employee WFC and job burnout [15]. In addition, Pinto et al. pointed out that high levels of work control and colleague support can mitigate the level of project staff burnout [50]. In the research conducted into the outcome variables of WFC in the field of construction engineering, Zhang et al. found that construction employees’ work and life satisfaction will decrease with an increase in WFC. In addition, WFC simultaneously impacts job performance [51]. Similarly, WFC has a significant impact on recovery needs as a mediator variable [16].

## 3. System Dynamics Model of the Evolution of Employee WFC in the Construction Industry

System dynamics is a method for modeling, simulating and analyzing complex systems [52]. The work-family life of employees in the construction industry is a complex dynamic system with broad range of interactions of different influencing factors. System dynamics can play a different role in probing the construction employee’s work-family system. The advantage of system dynamics is that considering aggregated variables encourages both a systemic view of the interactions of variables from the work and family domains, and a more strategic perspective of the management of the system. System dynamics provides a rigorous approach to examine the effects derived from the interaction of various processes [53], rather than look at factors in isolation. This paper constructs a bi-directional dynamic model framework of work-family conflict based on the literature review, and then establishes a system dynamics model of construction employee’s work-family conflict. Finally, a simulation of the model is conducted to draw conclusions.

### 3.1. Model Framework and Basic Hypothesis and Context

#### 3.1.1. Establishment of Model Framework

**Figure 1:**
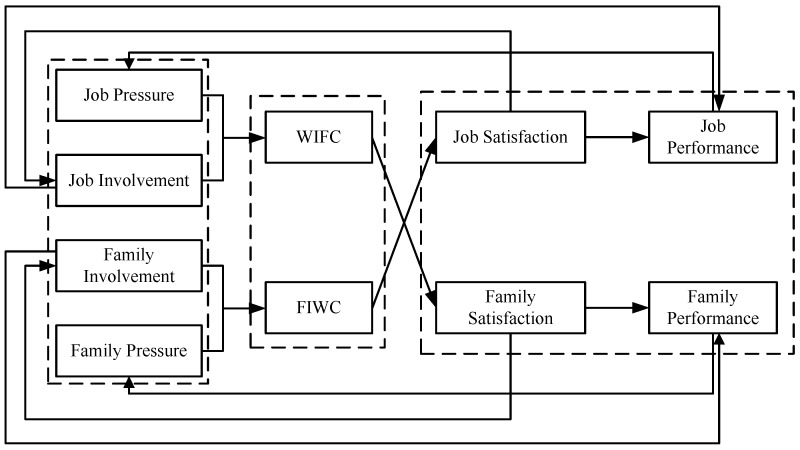
Bi-directional dynamic model framework of WFC.

Based on a critical review of existing WFC literature, this paper selects job pressure and job involvement from the work domain and family pressure and family involvement from the family domain as antecedent variables. WFC is divided into WIFC and FIWC, according to the bi-directional concept of WFC. In addition, we take job satisfaction, job performance, family satisfaction and family performance as the WFC outcome variables, in order to construct a bi-directional dynamic model framework of WFC evolution between work and family domains. The framework of the model is drawn as shown in Figure 1. In this model, job involvement and job pressure have a direct impact on WIFC, which directly affects family satisfaction. In turn, family satisfaction has a direct impact on family involvement on the one hand, and influences family pressure by impacting family performance on the other hand. Family involvement and family pressure both affect FIWC together. Similarly, FIWC can indirectly affect WIFC by having a direct impact on job satisfaction. In this way, the variables in our model from work and family domains form a bi-directional dynamic evolution relationship via the interaction between WIFC and FIWC.

#### 3.1.2. Basic Hypothesis and Context

A construction employee’s work-family life is a complex and ever-changing system which is affected by many factors. As a result, all the factors which may come into play are unlikely to be included in the model. The antecedents of WFC are mainly related to the three aspects of work domain, family domain and individual characteristics, while the outcome variables of WFC can be summarized into the three aspects of work-related, family-related and stress-related.

Existing studies in the academic community suggested that job pressure and job involvement were important antecedents, which were both positively correlated to WIFC. In addition, family pressure and family involvement were considered important antecedents, which were both positively correlated to FIWC [54,55]. The focus on job involvement and family involvement is the time spent working on the job and the time spent with family [56]. The variables of job pressure mainly include workload and job responsibility. Family pressure includes pressure from parents and spouses [23]. Accordingly, this paper posits the following hypothesis:

*Hypothesis 1*: Job involvement and job pressure from the work domain are positively related to WIFC, while family involvement and family pressure from the family domain are positively related to FIWC.

The individual characteristics of employees are important antecedents of WFC. An employee’s understanding of the relationship between work and family can be influenced by their values and beliefs. Aryee et al. pointed out that Chinese and Western employees see the relationship between work and family very differently [57]. Unlike in western cultures, Chinese culture tends to link the responsibilities of work and family to each other [58]. In China, a collectivism culture encourages employees to work hard. A career has been regarded as an important means of improving the quality of family life. In order to meet this need for individual career development, work is often given priority over family [59]. In contrast, in many western countries such as the United States, the employees often experience greater family demand, which will have greater impact on work-family conflict; allowing work to interfere with family is more likely to cause dissatisfaction in other family members that may lead to serious consequences [60]. To simplify our research, the study scope is limited to employees in the culture norm of collectivism. Therefore, this paper proposes the following hypothesis:

*Hypothesis 2*: Employees in this study have collective values such that their work life is more important than their family life. As such, the starting value of employee job satisfaction is greater than the starting value of family satisfaction.

WIFC has a significantly negative impact on family satisfaction, while FIWC has a significantly negative impact on job satisfaction [61]. The research conducted by Yang et al. reveals that job satisfaction has a significantly positive impact on employee job performance [62], while Luo pointed out that there is a positive correlation between family satisfaction and family performance [63]. Akhtar et al. proposed that job satisfaction and job involvement are significantly related [64], while Sukri et al. argued that job satisfaction can have a significant impact on job involvement [65]. Ma et al. confirmed that improving job involvement can improve the staff performance [66]. In the process of collecting literature, the authors did not find any relevant research into the direct relationship between family involvement and family performance. This may be due to the lack of any family performance-related scale development. In addition, family performance has not received enough attention from either management or organizational behavior researchers. However, from the perspective of theoretical analysis, the greater the family involvement and the more an employee takes on household tasks and shares more of the family responsibilities, the better will be that employee’s family performance. In theory, an improvement in job performance can alleviate employee job pressure, while an improvement in family performance can also alleviate family pressure. Therefore, the following hypotheses were put forward:

*Hypothesis 3*: WIFC is negatively correlated with family satisfaction, while FIWC is negatively correlated with job satisfaction.

*Hypothesis 4*: Job satisfaction is positively related to job performance and job involvement, while family satisfaction is positively related to family performance and family involvement.

*Hypothesis 5*: Job involvement is positively related to job performance, while job performance is negatively correlated with job pressure. Family involvement is positively related to family performance, while family performance is negatively correlated with family pressure.

WFC can specifically produce the three aspects of work-related, family-related and stress-related results. Apart from the work-related and family-related results, WFC can result in stress-related outcomes such as job burnout and emotional exhaustion as well. However, the work-family life of employees in the construction industry is a complex system, which involves numerous domains and influencing factors, it is therefore impossible to include all key variables in the model. This paper limits the outcome variables of WFC to the work-related results (job satisfaction and job performance) and the family-related results (family satisfaction and family performance). 

**Figure 2:**
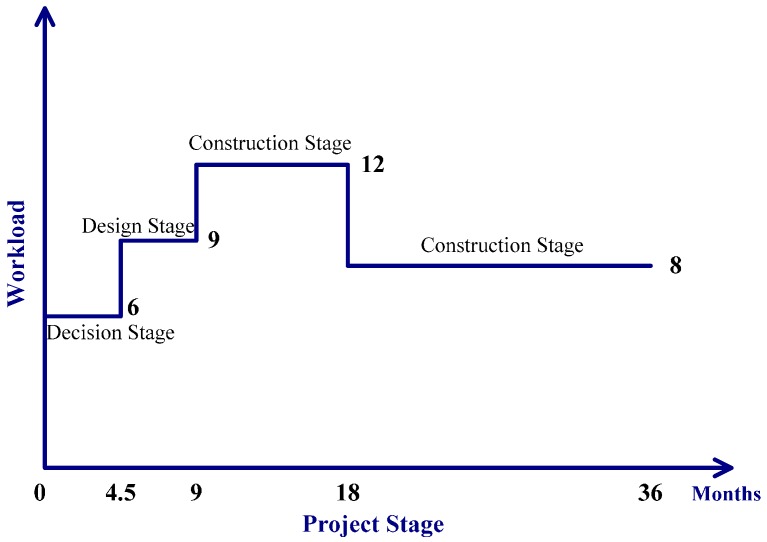
The staff workload during each stage of the construction project.

Based on the basic hypotheses above, the model context has been set as follows:

(1) The whole life cycle of the project can be divided into four stages, namely: (1) the decision stage; (2) the design stage; (3) the construction stage and (4) the operation and maintenance stage. Considering that the construction industry employees do not participate in the whole project lifecycle, it is assumed that they only participate in the first three stages. We set the decision stage time to be 4.5 months, the design stage time to be 4.5 months, the construction stage time at 27 months. The work situation of the construction industry employees is very complex. Some employees are “starters” and only work on the early tasks of the project before they go on to other projects, some are “finishers” and come from other projects to undertake completion works, this applies to supervisors as well as artisans. Similarly, some employees need to work for the project throughout the construction stage such as project managers, project technical principals and safety supervisors. In this paper, the study scope is limited to the employees who stay at the project in the whole construction stage.

(2) It is assumed that the job experience of the construction industry employees has a linear growth relationship with their working time as the project progresses. We set the initial value of job experience to be 0.5. At the end of the project, the value of job experience is 0.7.

(3) The workload of construction industry employees changes as the project progresses. We assume that the construction industry employees’ workload stays unchanged during the decision stage and the design stage (even if some of them do not participate in the project at these two stages, their workload in other projects keeps unchanged as well). The construction stage is divided into two phases. The employees’ workload in the first phase of the construction stage is greater than in the second phase of the construction stage, while it keeps unchanged in each of the two phases. The workload is set as shown in Figure 2. In Figure 2, the workload in each stage is described by some values without unit. The workload was set as being positively correlated to the working time.

### 3.2. WFC Evolution Causal Model

**Figure 3:**
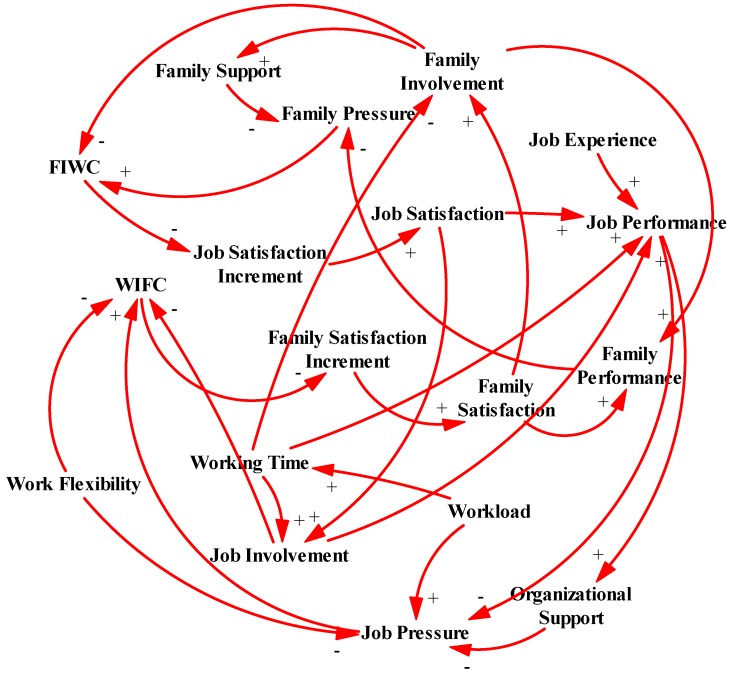
Causal relationship model for construction employee’s WFC evolution.

According to the model framework established proposed in the previous section, a causal relationship model for the construction employee’s WFC evolution was established by using the Vensim PLE software (Ventana Systems, Inc., Harvard, MA, USA) (see Figure 3).

As shown in Figure 3, WIFC affects family satisfaction through the influence of the family satisfaction increment. Conversely, FIWC affects job satisfaction by influencing the job satisfaction increment. Then, job satisfaction will have a positive impact on an employee’s job performance, and family satisfaction will have a positive impact on an employee’s family performance. In addition, job performance is affected by the employee’s job experience, working time and job involvement, while family performance is positively related to family involvement and family satisfaction. The causal relationship model presents system circles as follows:

- Family Involvement → FIWC → Job Satisfaction Increment → Job Satisfaction → Job Performance → Job Pressure → WIFC → Family Satisfaction Increment → Family Satisfaction → Family Involvement.
- Family Pressure → FIWC → Job Satisfaction Increment → Job Satisfaction → Job Performance → Job Pressure → WIFC → Family Satisfaction Increment → Family Satisfaction → Family Performance → Family Pressure.
- Job Pressure → WIFC → Family Satisfaction Increment → Family Satisfaction → Family Performance → Family Pressure → FIWC → Job Satisfaction Increment → Job Satisfaction → Job Performance → Job Pressure.
- Job Involvement → WIFC → Family Satisfaction Increment → Family Satisfaction → Family Performance → Family Pressure → FIWC → Job Satisfaction Increment → Job Satisfaction → Job Involvement, etc.

Furthermore, following the social support theory [6,67], the main source of social support in the field of WFC research is introduced in this paper: organizational support [30] and family support [6] as important moderators which influence WFC. In addition, work flexibility [32,33] from the boundary theory is also included in the causal relationship model, due to its relatively significant influence on WFC.

### 3.3. System Flow Diagram of the Construction Industry Employees WFC Evolution

**Figure 4:**
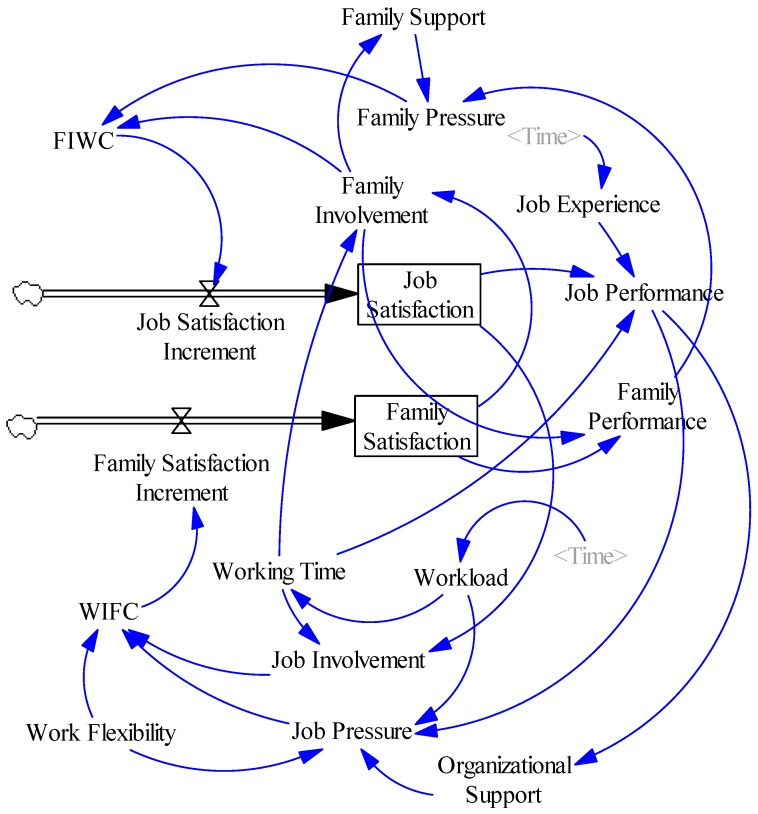
System flow diagram of construction industry employee WFC evolution. The diagram is constructed by the Vensim PLE software. There are two straight arrows in the system flow diagram which are called Rate. The Rate has a single arrowhead, indicating the direction that material can flow like a pipe with a valve. However, this is only a diagram, in a simulation model, the equation governs the direction that material can flow. In this system, the two rate variables flow to the two state variables as the material under the influence of FIWC and WIFC.

According to the causal relationship model (Figure 3), the system flow diagram of construction employee’s WFC evolution was established (see Figure 4).

As shown in Figure 4, there are 18 variables in the system: two state variables (job satisfaction and family satisfaction); two rate variables (job satisfaction increment and family satisfaction increment); 13 auxiliary variables (family support, family pressure, family involvement, job experience, job performance, family performance, workload, working time, job involvement, job pressure, organizational support, WIFC and FIWC) and one constant (work flexibility). The main equations (the functions in the equations below are derived from the Vensim PLE software) and design descriptions of the model are as follows:

Job satisfaction = INTEG (Job Satisfaction Increment, 5), the INTEG function performs numerical integration of job satisfaction increment starting at five.

Family satisfaction = INTEG (Family Satisfaction Increment, 1.5), the same as above, the INTEG function performs numerical integration of family satisfaction increment starting at 1.5.

Job experience = WITH LOOKUP (Time, ((0, 0)–(36, 1)), (0, 0.5), (36, 0.7)). The “WITH LOOKUP” is known as the table function which allows us to define customized relationships between a variable and its causes. The table function was used to represent the job experience of construction industry employees. To simplify the study, this paper has assumed that each employee’s job experience has a linear growth in the 36 units of simulation time, the initial value of job experience is 0.5 and the end value is 0.7.

Work flexibility = 1.

Job performance = working time × job experience × job involvement × DELAY1I (Job Satisfaction, 1, 5)/10. The DELAY1I function performs the first order exponential delay of job satisfaction for one month at the starting value of five. Considering that a change in job satisfaction will not immediately have a significant impact on job performance, the delay function is used here to reflect the delay of the influence of job satisfaction on job performance.

Family performance = DELAY1I (Family Satisfaction, 1, 1.5) × family involvement/10. As with the above, the DELAY1I function performs the first order exponential delay of family satisfaction for one month at the starting value of 1.5. Since the impact of family satisfaction on family performance also has a delay effect, the delay function is also used here. 

Family pressure = 1/(family support × family performance).

Family involvement = 100 × family satisfaction/working time.

FIWC = 0.05 × family involvement × family pressure.

WIFC = 0.05 × job involvement × job pressure/work flexibility.

Workload = WITH LOOKUP (Time, (((0, 0)–(36, 16)), (0, 6), (4.5, 6), (4.5, 9), (9, 9), (9, 12), (18, 12), (18, 8), (36, 8))). The table function was used to indicate the workload changes in the whole life cycle of the project.

Job involvement = job satisfaction × working time.

Job pressure = workload/(job performance × organizational support × work flexibility).

## 4. Model Simulation and Result Analysis

Model simulation can demonstrate logical argument or common sense derivation by means of visual interface in a transparent way. It can also help investigate the sensitivity of each variable to the change of the moderators in the system. The purpose of the model simulation is to test the validity and correctness of the simulation results. A validity test can verify whether or not the system of the reaction model reflects the rule changes and characteristics of the actual system. The analysis of the model can help correctly understand the precise problems which need to be analyzed. The main testing methods are the validity test of the model structure and behavior and the consistency test of the model structure and behavior with the actual system [68]. This model has been run in the Vensim PLE software (Ventana Systems, Inc., Harvard, MA, USA) and the simulation time was set to be 36 months.

### 4.1. Simulation Results under Given Parameters

**Figure 5:**
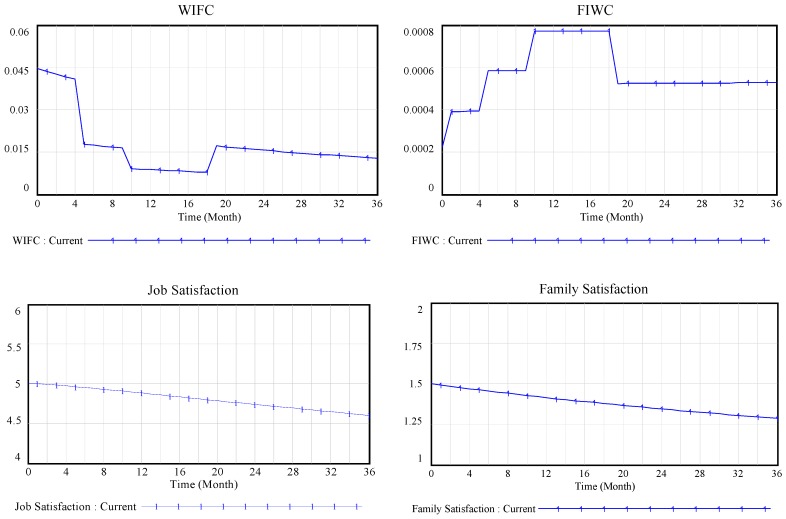

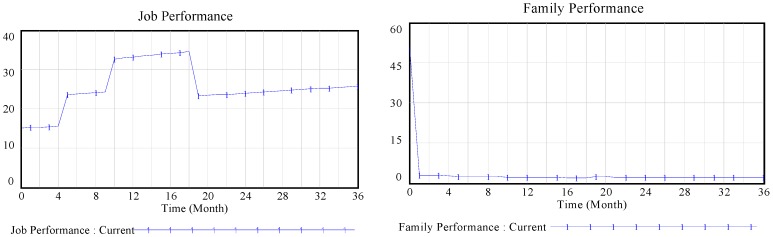
Simulation results under given parameters.

As shown in Figure 5, the employees always experience far greater WIFC than FIWC in the whole life cycle of the construction project. This is arguably due to that work and family play different roles in an employee’s life. The work-to-family interference is much greater than the family-to-work interference, given the effect of collectivism values. This finding is also consistent with the research of scholars such as Gutek and Netemeyer [23,25]. From the decision stage to the design stage to the first phase of the construction stage, FIWC shows a rising trend. The reason for this trend is that, as the project progresses, the employees’ workload continues to increase. The working time and job involvement subsequently increase, and then family involvement decreases, which in turn causes FIWC to rise. In addition, by the second phase of the construction stage, FIWC decreases with the reduction in the workload and the increase in the family involvement. WIFC shows a downward trend during the decision stage and the design stage and the first phase of the construction stage. This downward trend occurs, because, with the increase in job experience and working time, the employees’ job performance increases, thereby reducing the level of job pressure and improving the level of organizational support, at least to a certain extent. During the second phase of the construction stage, however, WIFC rebounds, due to the decline of employee job performance.

Directly affected by WIFC and FIWC, both family satisfaction and job satisfaction show a downward trend during the simulation time. However, due to the prevailing collectivism values, the level of WIFC is significantly higher than that of FIWC during the project, while the family satisfaction level is lower than the level of job satisfaction. By comparing job performance and family performance, the phenomenon can be observed whereby the employees’ job performance during different stages of the project is highly correlated to the working time. In addition, the overall growth trend of job performance is also related to the increase of job experience, while the level of family performance falls to a very low level in the early stage of the project, due to the dual influence of family involvement and family satisfaction. This fall in family performance is caused by low family involvement and low family satisfaction, because the employees pay more attention to work than their families.

### 4.2. Application Analysis of the Simulation Results

In order to determine the impact of the various parameters on job satisfaction, family satisfaction, job performance and family performance in the model, the parameter values of the variables in the model can be changed and the outputs can be compared. This paper chose to change the model’s parameter values of work flexibility, organizational support and family support. Consequently, the operating results of the model and the related parameters were compared.

**Figure 6:**
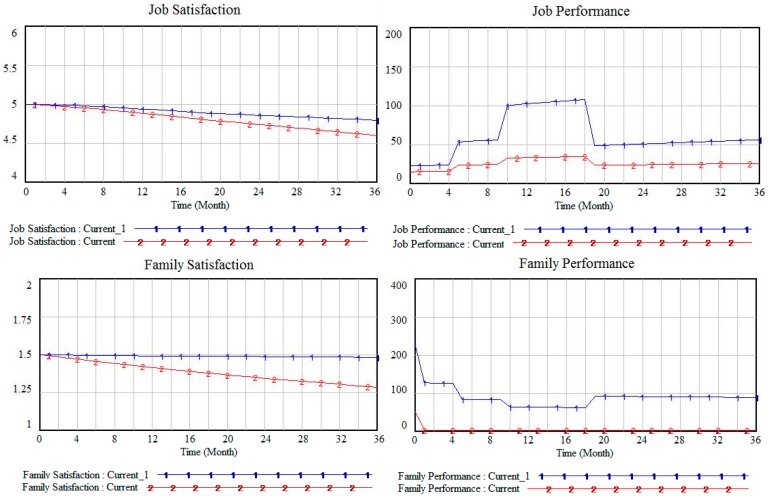
Satisfaction and performance under different work flexibility.

The work flexibility parameter value of the current scheme is 1. The other model parameters were left unchanged and raise the work flexibility parameter value to 5 in the Current_1 scheme, and the simulation results are shown in Figure 6.

By contrasting the results of Current_1 and Current in Figure 6, it can be observed that the employee job satisfaction and family satisfaction levels improve in the same simulation time when the level of work flexibility increases. In addition, both job performance and family performance also improve significantly, and the improvement in family performance is greater. Thus, it can be observed that the effect on employee satisfaction and performance caused by improved work flexibility is obvious. The main reason for this finding is that, when the level of work flexibility increases, the value of job pressure and WIFC reduces. The degree of family satisfaction then improves, and family involvement subsequently increases. The level of family performance increases significantly with the growth in family satisfaction and family involvement. This increase in family satisfaction greatly reduces family pressure, and then FIWC decreases. Since then, job satisfaction and job performance improves accordingly. In practice, an organization can offer employees flexible work arrangements, such as the provision of free time, setting up a home office or work sharing. These are just some of the ways to enhance work flexibility so that employees can more easily balance the relationship between work and family. This improved work-life balance will thus improve employee satisfaction and performance in their work and family domains.

**Figure 7:**
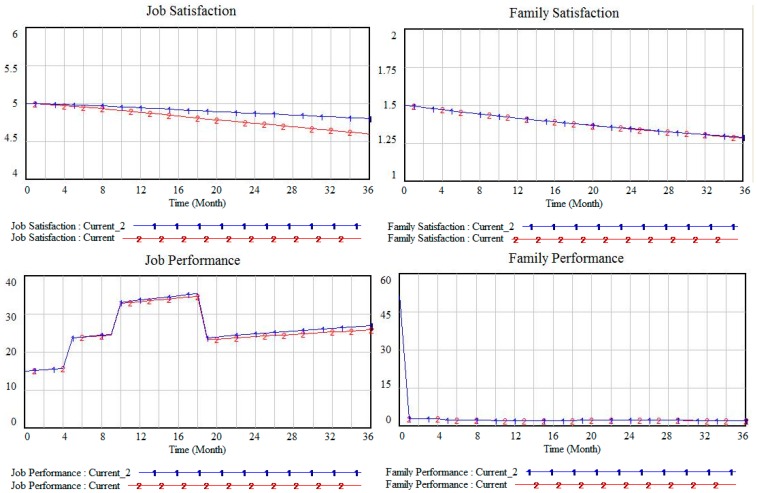
Satisfaction and performance under different family support.

In the original program Current, the family support function relationship is: Family support = family involvement × 1.0. All other parameters remained unchanged and only change the function relationship of family support to: Family support = family involvement × 2.0. In other words, the family support reaction degree to the family involvement was improved in order to get the scheme Current_2. The simulation results are shown in Figure 7. 

As shown in Figure 7, when improving the reaction degree of family support to family involvement (i.e., indirectly improving the level of construction industry employee family support), the degree of family satisfaction and family performance from the employee family domain basically do not change. However, the levels of job satisfaction and job performance from the employee work domain show a certain degree of improvement. Compared to job satisfaction, the improvement in job performance is relatively insignificant. This finding indicates that increasing the level of family support can improve job satisfaction and job performance, at least to a certain extent. At the same time, increased family support will apparently have very little influence on the employee’s satisfaction and performance within the family domain. This finding can be attributed to the result whereby after the changes to the family support function relationship, employee family pressure drops directly, thereby causing FIWC to decline. Thus, the degree of job satisfaction goes up, and job performance also subsequently improves. The improvement in job performance is relatively insignificant, because family support only indirectly affects job performance through FIWC’s influence on job satisfaction. Family pressure cannot directly affect family satisfaction and family performance. Job satisfaction and job performance can only indirectly affect family satisfaction and family performance through such influencing factors such as job involvement, job pressure and organizational support. Due to the fact that there are too many parameters in the influence path, family satisfaction and family performance hardly change.

**Figure 8:**
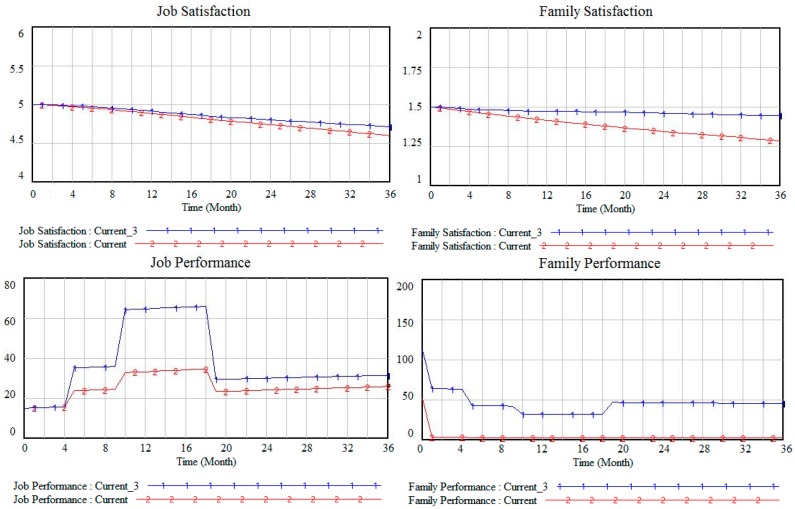
Satisfaction and performance under different organizational support.

In the original program Current, the organizational support function relationship is: Organizational support = job performance × 0.1. All other parameters remained unchanged and only change the function relationship of organizational support to: Organizational support = job performance × 0.3 (i.e., the organizational support reaction degree to job performance was improved) to get the scheme Current_3. The simulation results are shown in Figure 8. 

Figure 8 shows that, when raising the reaction degree of organizational support to job performance, (i.e., indirectly improving organizational support), job satisfaction, job performance, family satisfaction and family performance all improve accordingly. However, the improvement in terms of job satisfaction is not significant when compared with family satisfaction. In addition, the improvement in job performance is not as obvious as the improvement in family performance. The reason for this finding is that improvement in organizational support leads to a reduction of employee job pressure, which in turn causes family satisfaction to improve significantly (by reducing WIFC). Family involvement subsequently increases, while family performance obviously improves under the joint function of family satisfaction and family involvement. As such, family pressure levels drops substantially. Then, FIWC decreases. Thus, job satisfaction and job performance experience a corresponding increase. In reality, the organization/employer can improve organizational support by improving the work environment and strengthening humanistic care, in order to improve employee satisfaction and performance. 

## 5. Conclusions

The work-family conflict has attracted a growing public awareness. The topic is one of great concern to scholars from the fields of management and psychology. Although research methods are increasingly diversified, the mainstream research methods are quantitative, e.g., questionnaire surveys. Through extensive literature review, a bi-directional dynamic model framework is developed for the work-family conflict of employees in the construction industry. Based on the method of system dynamics, an evolution model of employee’s work-family conflict in the construction industry was developed. This model includes the introduction of several key antecedents and consequences of WFC. The simulation results of the model indicate the following: (1) Construction employees of collective values experience WIFC to a significantly greater degree than FIWC; (2) Improving work flexibility and organizational support can have a positive impact on employee satisfaction and performance from the perspective of work and family domains; (3) Improving family support can only significantly increase job satisfaction. Therefore, in practice, the organization could arrange for their employees to have a flexible work plan. Another option would be to improve the organizational climate as a means to improve the degree of employee satisfaction and performance levels. In addition, an employee’s family members can improve that employee’s satisfaction levels by providing improved family support, such as instrumental assistance and emotional care.

This model can explain the evolution of construction employee’s work-family conflict, at least to some degree. However, since WFC involves numerous domains and influencing factors, it is not possible to include all key variables in the model. Hence, our model is not necessarily a true reflection of the actual system. In future research, the introduction of additional model parameters (e.g., the stress-related outcome variables) for analysis should be considered as a means to further our research conclusions and provide more universal significance. In addition, considering that individuals operate within a number of domains which exist beyond work and family (e.g., social community), additional research is required to involve other domains in which the individuals inhabit. Furthermore, some qualitative research methods, such as case studies, content analysis, grounded theory, etc. could also be introduced.
